# Mindfulness Interventions on Mental Health in Working Adults: A Scoping Review

**DOI:** 10.3390/healthcare14050621

**Published:** 2026-02-28

**Authors:** Edgar Vásquez-Carrasco, Vanesa Bruna, Javiera Barraza, Nayaret Escobar, Lorena Sepúlveda, Francisca Bastidas, Benjamín González, Jordan Hernandez-Martinez, Cristian Sandoval-Vásquez, Eduardo Carmine-Peña, Constanza Lorca, Celia Sánchez-Gómez, Pablo Valdés-Badilla, Pamela Marcone-Dapelo

**Affiliations:** 1 Faculty of Psychology, School of Occupational Therapy, Universidad de Talca, Talca 3465548, Chile; edgar.vasquez@utalca.cl; 2 Centro de Investigación en Ciencias Cognitivas, Facultad de Psicología, Universidad de Talca, Talca 3465548, Chile; 3 VITALIS Longevity Center, Universidad de Talca, Talca 3465548, Chile; 4 Carrera de Terapia Ocupacional, Facultad de Ciencias de la Salud, Universidad de Playa Ancha, Valparaíso 2360072, Chile; vanesa.bruna@alumnos.upla.cl (V.B.); javierabarraza@alumnos.upla.cl (J.B.); nayaret.escobar@alumnos.upla.cl (N.E.); lorena.sepulveda@alumnos.upla.cl (L.S.); francisca.lagos@alumnos.upla.cl (F.B.); b_gonzalez@alumnos.upla.cl (B.G.); 5 Department of Physical Activity Sciences, Universidad de Los Lagos, Osorno 5290000, Chile; jordan.hernandez@ulagos.cl; 6 Facultad de Salud, Escuela de Tecnología Médica, Universidad Santo Tomás, Los Carreras 753, Osorno 5310431, Chile; cristian.sandoval@ufrontera.cl; 7 Departamento de Medicina Interna, Facultad de Medicina, Universidad de La Frontera, Temuco 4811230, Chile; 8 Carrera de Medicina, Facultad de Medicina, Universidad de La Frontera, Temuco 4811230, Chile; e.carmine01@ufromail.cl; 9 Departamento de Ciencias Básicas, Facultad de Medicina, Universidad de La Frontera, Temuco 4811230, Chile; 10 Department of Developmental and Educational Psychology, University of Salamanca, 37008 Salamanca, Spain; celiasng@usal.es; 11 Institute of Biomedical Research of Salamanca (IBSAL), 37007 Salamanca, Spain; 12 Department of Physical Activity Sciences, Faculty of Education Sciences, Universidad Católica del Maule, Talca 3530000, Chile; 13 Sports Coach Career, Faculty of Life Sciences, Universidad Viña del Mar, Viña del Mar 2520000, Chile; 14 Laboratorio de Investigación en Salud, Departamento de Rehabilitación, Intervención y Abordaje Terapéutico, Universidad de Playa Ancha, Valparaíso 2360072, Chile

**Keywords:** adults, health status, rehabilitation, works

## Abstract

**Background**: This scoping review aimed to identify and synthesize the evidence on mindfulness-based interventions targeting mental health outcomes in working adults. **Methods**: A comprehensive search was conducted in four electronic databases (PubMed, Scopus, Web of Science, and OTseeker) up to October 2025. The review followed PRISMA-ScR guidelines. Methodological quality was evaluated using the Oxford Centre for Evidence-Based Medicine (OCEBM) classification. The protocol was prospectively registered on the Open Science Framework (OSF). **Results**: A total of 1803 records were identified, of which nine randomized controlled trials met the inclusion criteria. The included studies examined Mindfulness-Based Stress Reduction, Mindfulness-Based Self-Care, Mindfulness-Oriented Therapy, and digital mindfulness interventions. Overall, mindfulness interventions demonstrated beneficial effects across several mental health domains. **Conclusions**: Mindfulness-based interventions show promising benefits for improving mental health among working adults. Their structured, brief, and adaptable formats support their feasibility for integration into occupational health programs and workplace mental health promotion.

## 1. Introduction

Mindfulness refers to the ability to maintain intentional, present-centered awareness of one’s experiences, observing them openly and non-judgmentally [1]. Originating in Eastern contemplative traditions, mindfulness has been adapted to Western clinical settings to address various mental and physical health conditions [1]. Its integration into clinical practice has been associated with improvements in mental health, chronic pain management, and overall quality of life [2,3]. Regular mindfulness practice has been linked to significant reductions in stress, anxiety, and depression, as well as enhanced emotional regulation and body awareness [4,5,6]. These benefits are thought to result from mechanisms such as attention regulation, body awareness, emotional regulation, and positive changes in self-perception [7].

Structured mindfulness-based programs including Mindfulness-Based Stress Reduction (MBSR), Mindfulness-Based Cognitive Therapy (MBCT), and Mindfulness-Based Occupational Therapy (MBOT), have shown effectiveness across a wide range of populations, particularly among individuals experiencing chronic pain, anxiety, depression, or elevated stress levels [8,9]. In addition to these well-established approaches, newer applications of mindfulness highlight its potential to move beyond internal awareness and support meaningful participation in daily activities, ultimately contributing to improved cognitive functioning and mental well-being [10]. When mindfulness principles are incorporated into occupational performance, therapeutic efforts focus not only on reducing psychological distress but also on promoting greater involvement, engagement, and satisfaction in everyday routines [11].

This integrative perspective is consistent with person-centered occupational therapy principles, which prioritize autonomy, empowerment, and self-directed participation in meaningful activities [12]. In contrast to conventional mindfulness programs such as MBSR and MBCT, MBOT explicitly addresses functional performance and contextual demands. By targeting these dimensions, MBOT aims to enhance psychological regulation and support more effective engagement in everyday occupations [10,13].

Research increasingly demonstrates that mindfulness-based therapies can effectively reduce symptoms of anxiety and depression, as well as enhance cognitive performance and overall quality of life [14]. Positive outcomes have been observed in populations with musculoskeletal disorders, chronic pain, and neurological conditions, where mindfulness practices promote functional recovery and adaptation to daily challenges [14,15]. Mindfulness therapies are linked to improved mental health and emotional well-being in adults [16]. Despite the growing literature on mindfulness-based interventions and mental health outcomes, the evidence remains heterogeneous in terms of intervention formats, outcomes, occupational contexts, and study designs. This variability, together with inconsistent findings across studies, limits the feasibility of a focused systematic review or meta-analysis; therefore, a scoping review is warranted to qualitatively map and contextualize the existing evidence. This scoping review seeks to identify and synthesize current research on mindfulness-based approaches as MBSR, MBCT, and MBOT and their effects on mental health among working adult populations.

## 2. Materials and Methods

### 2.1. Protocol and Registration

This scoping review was conducted in accordance with the Cochrane Collaboration guidelines [17] and reported following the PRISMA-ScR framework, including both the checklist and the flow diagram [18]. The protocol was prospectively registered on the Open Science Framework (OSF) (available on: https://osf.io/uprhz/overview, accessed on 24 November 2025).

### 2.2. Eligibility Criteria

This scoping review included peer-reviewed original studies, specifically randomized controlled trials (RCTs), with no restrictions on language or publication date, up to October 2025. Exclusion criteria comprised conference abstracts, books, book chapters, editorials, letters to the editor, protocols, reviews, case reports, and non-randomized studies. Study selection was guided by the PICOS (Population, Intervention, Comparator, Outcome, Study design) framework, as detailed in Table 1.

### 2.3. Information Search Process and Database

An electronic search was conducted across four databases: Scopus, Web of Science (Core Collection), MEDLINE/PubMed, and OTseeker. The search strategy integrated Medical Subject Headings (MeSH) from the U.S. National Library of Medicine with relevant keywords related to mindfulness interventions, mental health, and working adults. The Boolean string included terms such as: (“Working Adults” OR “Employed Adults” OR “Adult Workers” OR “Employees” OR “Professionals” OR “Workforce” OR “Labor Force” OR “Working Population” OR “Workers”) AND (“Mindfulness” OR “Mindfulness Meditation” OR “Mindfulness-Based Stress Reduction” OR “Mindfulness-Based Cognitive Therapy” OR “Mindfulness-Based Intervention” OR “Mindfulness Training” OR “Meditation, Mindfulness” OR “Mindfulness-Based Programs” OR “MBSR” OR “MBCT” OR “Mindfulness Practices”) AND (“Adults” OR “Adult Population” OR “Adult Subjects” OR “Young Adults” OR “Middle-Aged Adults”).

Two independent experts assessed the eligibility of the retrieved articles based on predefined inclusion and exclusion criteria. Both reviewers held doctoral degrees in health-related sciences and had peer-reviewed publications indexed in Journal Citation Reports^®^. To reduce potential selection bias, the reviewers were not informed of the search strategy. A final search update was performed on 30 October 2025, to identify relevant errata or retractions associated with the included studies.

### 2.4. Study Selection Process and Data Collection

All studies were exported to Zotero Reference Manager (Version 7.0.30, Roy Rosenzweig Center for History and New Media, George Mason University, Fairfax, VA, USA), and the selection process is illustrated in the PRISMA flow diagram. Three researchers (V.B., J.B. and N.E.) and an expert in the field (E.V.-C.) independently conducted the screening process. Duplicates were removed using Zotero. Initially, titles and abstracts of all potentially eligible studies were reviewed, followed by a full-text assessment according to the predefined inclusion criteria. Articles meeting the eligibility requirements and reaching consensus among all reviewers were included in the final analysis.

### 2.5. Methodological Quality Assessment

Methodological quality and level of evidence were appraised using the Oxford Centre for Evidence-Based Medicine criteria [19]. This appraisal was conducted to classify the evidence level of the included studies. According to the predefined eligibility criteria, only randomized controlled trials classified as Level 1b were included, while studies at lower evidence levels (Levels 2–5) were excluded. This assessment was used for descriptive purposes and did not influence data synthesis or interpretation.

### 2.6. Data Synthesis

Data from the included studies were extracted into a standardized Excel^®^ spreadsheet (version 16.81; Microsoft Corporation, Redmond, WA, USA), following Cochrane methodological recommendations [17]. Three reviewers (V.B., J.B. and N.E.) independently performed data extraction and verified consistency. Extracted variables included: (i) author and year of publication, (ii) country of origin, (iii) study design, (iv) intervention tools, (v) number of participants per group, (vi) intervention program details, (vii) outcome measures (mental health), and (viii) main results.

## 3. Results

### 3.1. Included Studies

**Figure 1:**
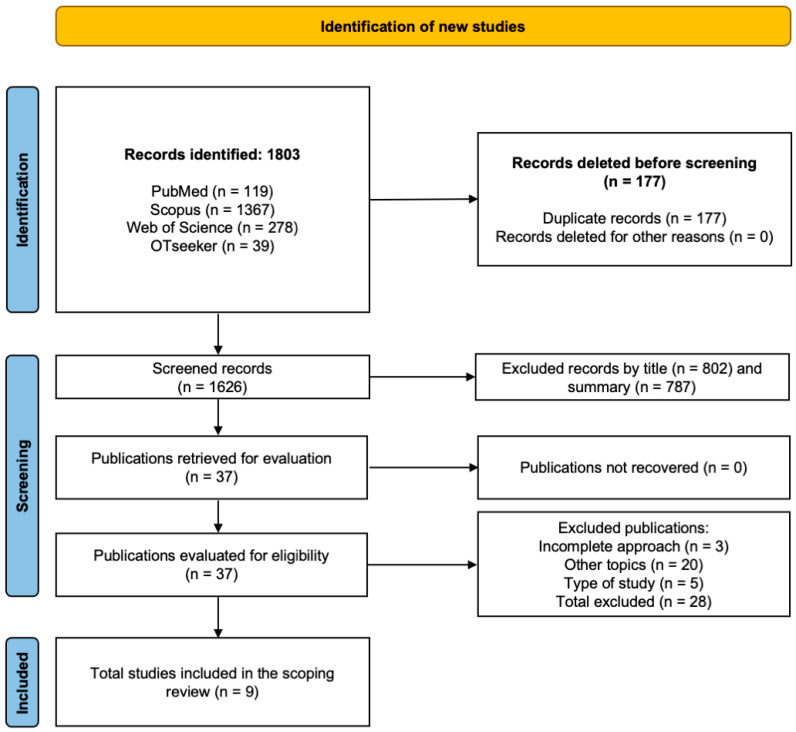
Flowchart of the scoping review [29].

A total of 1803 records were identified through the database search, of which 177 were duplicates. After removing duplicates, 1626 records remained, of which 1589 were excluded following title screening (n = 802) and abstract screening (n = 787) due to lack of relevance. The full-text review of 37 articles resulted in the exclusion of 28 studies that did not meet the inclusion criteria: 3 employed incomplete methodologies, 20 addressed unrelated topics, and 5 used an incompatible study design. Ultimately, 9 studies met all criteria and were included in the scoping review (Figure 1) [20,21,22,23,24,25,26,27,28].

**Figure 2:**
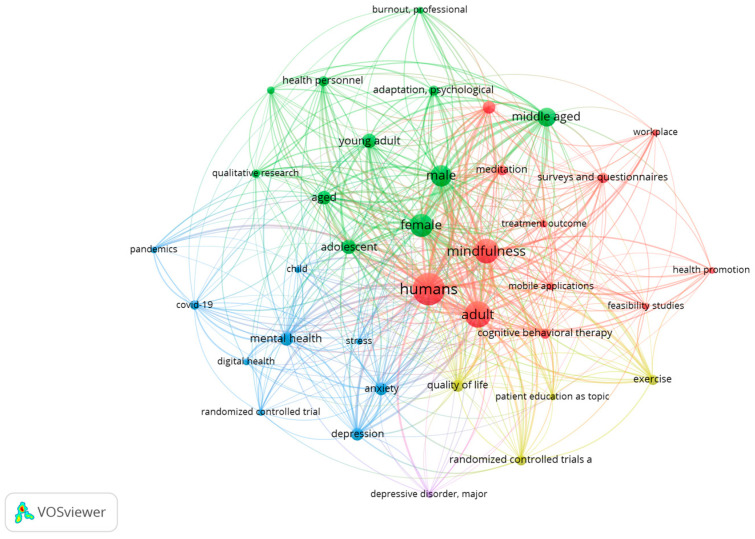
Topical distribution of the literature on mindfulness interventions on mental health in working adults.

**Figure 3:**
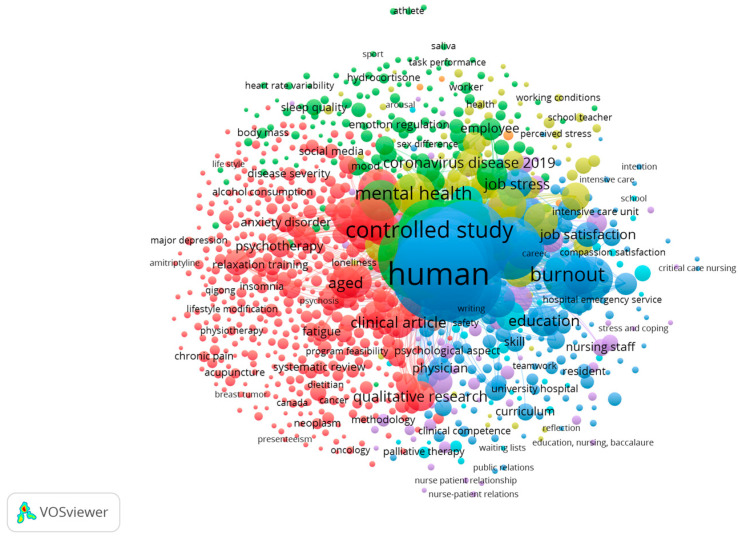
The literature on specific intervention types (e.g., MBSR, mobile-based programs, cognitive-behavioral strategies) and associated outcomes.

All included studies evaluated mindfulness-based interventions or related strategies aimed at reducing stress, enhancing psychological well-being, or fostering psychological detachment among healthcare professionals and other high-demand workers. Table 2 summarizes the evidence provided by these articles in relation to the research questions and PICOS standards. The evidence, comprising randomized clinical trials and pilot studies published between 2018 and 2024, reflects the contemporary relevance and rapid development of this research area. The database with the largest number of records was Scopus. A thematic map was generated using VOSviewer (version 1.6.20) to depict the conceptual structure of mindfulness-based interventions and their reported effects on stress, burnout, cognitive function, and mental health. Figure 2 presents the overarching thematic organization of these interventions, while Figure 3 illustrates the clustering of specific intervention types (e.g., MBSR, mobile-based programs, cognitive-behavioral strategies) and associated outcomes. Both figures were derived from the selected descriptors and their co-occurrence patterns.

### 3.2. Methodological Quality

The methodological quality of the studies included in this scoping review is considered moderate to high. All selected studies employed RCT designs, representing a strong level of methodological rigor consistent with the Oxford Centre for Evidence-Based Medicine level 1b classification [20,21,22,23,24,25,26,27,28].

### 3.3. Characteristics of the Studies and Interventions

Nine randomized controlled trials were conducted in working or professional adult populations to evaluate the effects of mindfulness-based interventions. The study samples encompassed a wide range of occupational groups, including healthcare professionals, nurses, teachers, military recruits, and employees from corporate and public sectors, with participant ages ranging from 18.9 to 59 years. The trials were carried out across multiple international settings such as the United States, the United Kingdom, Chile, China, Germany, Iran, Japan, and Türkiye, reflecting diverse cultural and occupational contexts.

Intervention durations ranged from 1 to 8 weeks, typically involving one session per week, with individual sessions lasting between 10 and 120 min. The interventions employed various mindfulness-based protocols, including MBSR, MBSC, MBOT, app-based meditation (e.g., Headspace^®^), psychoeducational stress management programs, and mindfulness combined with music therapy.

### 3.4. Mental Health

Mindfulness interventions generally showed beneficial effects across multiple dimensions of mental health. Several studies reported significant reductions in perceived stress [20,21,23,24], suggesting potential improvements in emotional self-regulation and coping with work-related stressors. Likewise, reductions in symptoms of anxiety and depression were observed in some studies [21,27], accompanied by improvements in overall psychological well-being [21,28].

However, findings related to professional burnout and general mental health outcomes were more heterogeneous. While some studies reported reductions in burnout [25] and improvements in general mental health indicators [23], others found no statistically significant changes [20,22,26], indicating variability in intervention effects across different populations and settings. These mixed results suggest that the impact of mindfulness-based interventions may be influenced by factors such as intervention format, duration, participant characteristics, and occupational context.

Overall, the available evidence suggests that mindfulness-based interventions may contribute to improvements in psychological well-being and stress-related outcomes in occupational and educational contexts. Nevertheless, these findings should be interpreted with caution, as most interventions were of relatively short duration and included limited follow-up periods, restricting conclusions regarding the sustainability of observed effects. Furthermore, the cultural, occupational, and contextual heterogeneity across the included studies limits the generalizability of the findings and underscores the need for future research with longer follow-up periods and more context-sensitive designs.

### 3.5. Adverse Effects and Adherence

Adherence averaged 93% across the nine trials, and no adverse effects were reported. These findings suggest that mindfulness-based interventions are both feasible and well tolerated in adult working populations, reinforcing their potential for broader implementation in occupational and clinical settings.

## 4. Discussion

Based on the findings of this scoping review, mindfulness-based interventions demonstrate positive effects across multiple mental health domains. Evidence indicates significant reductions in perceived stress, improved emotional regulation, and enhanced capacity to cope with work-related demands. Reductions in anxiety and depressive symptoms have also been reported, reflecting improved psychological functioning and greater resilience. These short-term effects represent key indicators of enhanced mental well-being within the populations studied.

### 4.1. Mental Health

A randomized controlled trial involving individuals with anxiety and depression indicated that a MBOT program resulted in significant and sustained improvements in subjective well-being (*p* < 0.01) over a nine-week period. The observed effects correlated with heightened activity in the left dorsolateral prefrontal cortex (*p* < 0.02), indicating its potential role in enhancing social participation and occupational performance [26]. Alfuth et al. [30] reported similar outcomes, noting significant improvements in occupational performance and satisfaction in self-care, productivity, and leisure domains (*p* < 0.001), along with a moderate increase in concentration (*p* < 0.001). The findings indicate that mindfulness and perception-based approaches can yield quantifiable occupational benefits for individuals with mental health conditions, highlighting their significance in occupational therapy practice.

A meta-analysis examining mindfulness- and acceptance-based interventions in individuals with multiple sclerosis reported substantial reductions in depression, anxiety, stress, and pain, along with moderate improvements in quality of life [31]. Although these findings are promising, they should be interpreted with caution and not considered as a stand-alone intervention. Research at the intersection of mindfulness and occupational therapy remains limited, highlighting the need for further studies given its potential clinical applicability and social relevance. Mindfulness enhances present-centered attention, a process associated with cognitive, emotional, and interpersonal benefits that may indirectly facilitate physical functioning and greater autonomy [32,33]. Improvements in these domains may reduce the risk of adverse events, such as falls, thereby preventing related injuries and enhancing overall quality of life [34,35]. The presence of non-significant results in several outcomes limits the strength of the conclusions and reinforces the exploratory nature of the available evidence.

A major challenge in strengthening the scientific validation of mindfulness lies in the need for rigorous research designs, including larger sample sizes and methodological advances such as the use of neuroimaging techniques [2]. Regular mindfulness practice has been associated with multiple benefits, including reductions in stress and anxiety, increased emotional awareness, improved emotional regulation, and stronger interpersonal relationships [36]. A solid theoretical framework is essential to understand this complexity, as neurobiological evidence indicates structural and functional changes in brain regions involved in attention and emotion regulation, including the prefrontal cortex, insula, hippocampus, amygdala, and the default mode network [37].

In occupational settings, mindfulness has been associated with reduced work-related stressors that negatively affect engagement [2]. Moreover, increases in cortical thickness in regions related to emotional regulation have been documented, leading to improved cognitive performance and reductions in anxiety and depressive symptoms [38]. Mindfulness has also demonstrated greater efficacy than placebo in pain management, possibly due to its influence on the orbitofrontal cortex and anterior cingulate cortex [38].

### 4.2. Limitations and Strengths

The present scoping review has several limitations: (i) methodological heterogeneity in intervention duration, frequency, and session length, hindering direct comparisons; (ii) variable sample sizes, from small pilot trials (<30 participants) to medium-scale studies (>100 participants), limiting representativeness; (iii) occupational diversity among participants (healthcare, education, military, corporate), affecting generalizability; (iv) heterogeneous assessment tools for psychological and occupational outcomes, complicating synthesis; and (v) short follow-up periods, preventing evaluation of long-term effects. Nevertheless, this review also presents key strengths: (i) inclusion of randomized controlled trials, ensuring methodological rigor within a scoping framework; (ii) comprehensive mapping of diverse mindfulness interventions modalities (MBSR, MBSC, MBOT, app-based, and music-assisted); (iii) consistent positive trends in mental health and occupational well-being, with high adherence (93%) and no adverse events, confirming feasibility; and (iv) incorporation of physiological and neurobiological measures, expanding understanding of mindfulness mechanisms relevant to occupational therapy.

### 4.3. Practical Applications

From an occupational therapy perspective, the findings of this exploratory review highlight the increasing integration of mindfulness practices as supportive interventions for work-related stress, emotional regulation, and professional well-being. Mindfulness interventions can be incorporated into disciplinary models to improve attention, self-awareness, and coping strategies in individuals exposed to work-related stress. These results also suggest that short-term, structured programs whether face-to-face, digital, or hybrid can be feasibly implemented in healthcare, educational, and corporate settings. Interprofessional collaboration among occupational therapists, psychologists, and human resources professionals can optimize the adaptation of mindfulness protocols to the specific needs of different work environments. Furthermore, mindfulness-based approaches could serve both preventative and rehabilitative purposes, fostering resilience and productivity in demanding work contexts.

### 4.4. Research and Policy Implications

At the research level, this review highlights the need for broader empirical efforts to strengthen the evidence base for mindfulness interventions. Future studies should include larger and more diverse samples, standardized protocols, and longitudinal follow-up to examine the maintenance of effects. Incorporating neurobiological, psychophysiological, and performance-based outcomes will enhance understanding of underlying mechanisms and occupational relevance.

At the policy level, the findings underscore the potential of integrating mindfulness-based interventions into organizational health promotion strategies. Implementing these interventions as part of occupational wellness policies may contribute to stress reduction, improved job satisfaction, and reduced absenteeism. Developing cost–benefit analyses and implementation studies will be essential to evaluate feasibility and scalability across different professional sectors and healthcare systems.

## 5. Conclusions

Mindfulness-based interventions showed overall positive effects on mental health, particularly in reducing perceived stress, anxiety, and depressive symptoms while enhancing psychological well-being. Overall, longer and sustained mindfulness practices appeared to yield greater benefits for psychological well-being in occupational contexts. However, methodological heterogeneity and limited follow-up durations indicate the need for more rigorous and standardized research. Expanding high-quality studies will be key to clarifying the role of mindfulness within occupational therapy practice and its broader applications in promoting mental health, resilience, and engagement in the workplace.

## Figures and Tables

**Table 1:** Selection criteria used in the scoping review.

Criteria	Inclusion Criteria	Exclusion Criteria
Population	Studies were included if they involved working adult participants, either with a mean age of 18 years or older.	Studies focusing on non-working populations, unless general adult population is included
Intervention	Studies involving mindfulness (Mindfulness-Based Stress Reduction, Mindfulness-Based Cognitive Therapy, and Mindfulness-Based Occupational Therapy) interventions lasting four weeks or more.	Studies that include other types of complementary interventions, not related to mindfulness Interventions.
Comparison	Interventions with active or inactive control groups.	Lack of baseline and/or follow-up data. Absence of control group.
Outcomes	Mental health (i.e., stress, anxiety and burnout) assessments	Lack of baseline data and/or follow-ups.
Study design	Randomized controlled trials, with pre- and post-assessment.	Controlled, retrospective, prospective and cross-sectional, non-randomized studies.
Level of evidence	1a	1b, 2a, 2b, 3a, 3b, 4 and 5

**Table 2:** Selected studies on mindfulness interventions on mental health in working adults.

References	Country	Study Design	Initial Sample	Average Age (Years)	Type of Mindfulness Interventions	Training Volume			Mental Health Assessments	Outcomes
						Week	Frequency (Sessions/Weeks)	Session Duration (min)		
[20]	USA	RCT	Health professionals from the NIH, no serious conditions	28	Mindfulness- based self -care	5	1	90	PSS-10, VAS-A and MBI	↑ PSS-10 (*p* = 0.02) ↑ VAS-A (*p* = 0.001) ↔ MBI (*p* = 0.99)
[21]	UK	RCT	Adult employees without illnesses from two large companies, one pharmaceutical and one technology	36 years	App-based mindfulness meditation (Headspace^®^)	8	1	10 to 20	WBS and HADS	↑ WBS (*p* = 0.003) ↑ Anxiety Scale (*p* = 0.033) ↑ Depression Scale (*p* = 0.065)
[22]	CL	RCT	Adult non-medical health workers in direct contact with patients, without suicidal ideation or problematic alcohol use	40 years	Psychoeducational course on stress management with theoretical classes and experiential activities	8	1	120	GHQ-12, PSS and job satisfaction	↔ GHQ-12 (*p* = 0.68) ↔ PSS (*p* = 0.14) ↔ Job Satisfaction (*p* = 0.63)
[23]	CN	RCT	49 Chinese military recruits (young adults) with psychological distress	19	MBSR	8	1	120	GHQ-12 and PSS-10	↑ GHQ-12 (*p* = 0.01) ↔ PSS-10 (*p* = 0.05)
[24]	DE	RCT	Profile: Working adults, mostly teachers or public service employees	29 years	Individual digital intervention, although participants were assigned to experimental groups (mindfulness, cognitive–behavioral or control)	6	1	25–60	PSS	↑ PSS (*p* = 0.001)
[25]	IR	RCT	Nurses working in geriatric wards	30 years	MBSR	8	1	120	MBI	↑ MBI (*p* = 0.001)
[26]	JP	RCT	29 adults	41 years	MBSR-OT	8	1	90	HAM-D	↔ HAM-D (*p* = 0.25)
[27]	CN	RCT	Young and middle-aged adults (active workers in psychiatric hospitals)	29	MBSR	8	1	60	SCL-90 scale, SDS, Self-Rating Anxiety Scale	↑ SCL-90 scale (*p* = 0.001) ↑ SDS (*p* = 0.001) ↑ Self-Rating Anxiety Scale *p*: 0.001
[28]	TK	RCT	Healthy young adult nurses exposed to occupational stress	28 years	Mindfulness-based conscious breathing + music therapy	1	1	30	State anxiety inventory, job strain scale and psychological well-being scale	↔ State anxiety inventory (*p* = 0.10) ↔ Job strain scale (*p* = 0.30) ↑ Psychological well-being scale (*p* = 0.036)

CL: Chile; CN: China; DE: Germany; GHQ-12: General Health Questionnaire—12; HADS: Hospital Anxiety and Depression Scale; HAM-D: Hamilton Rating Scale for Depression; IR: Iran; JP: Japan; MBI: Maslach Burnout Inventory; MBSR: Mindfulness-Based Stress Reduction; MBSR-OT: Mindfulness-Based Occupational Therapy; NIH: National Institutes of Health; PSS: Perceived Stress Scale; PSS-10: Perceived Stress Scale—10 items; RCT: Randomized Controlled Trial; SCL-90: Symptom Checklist—90; SDS: Self-Rating Depression Scale; TK: Turkey; UK: United Kingdom; USA: United States of America; VAS-A: Visual Analog Anxiety Scale; WBS: Well-being Scale; ↑: indicates a statistically significant improvement/increase in the reported outcome; ↔: no significant difference in the reported outcome.

## Data Availability

Data sharing is not applicable to this article as no new data were created or analyzed in this study.
